# The Prognostic Role of Candidate Serum Biomarkers in the Post-Acute and Chronic Phases of Disorder of Consciousness: A Preliminary Study †

**DOI:** 10.3390/brainsci14030239

**Published:** 2024-02-29

**Authors:** Rita Formisano, Mariagrazia D’Ippolito, Marco Giustini, Sheila Catani, Stefania Mondello, Iliana Piccolino, Filomena Iannuzzi, Kevin K. Wang, Ronald L. Hayes

**Affiliations:** 1 Neurorehabilitation 2, Post-Coma Unit, IRCCS Fondazione Santa Lucia, 00179 Rome, Italy; mg.dippolito@hsantalucia.it; 2 Environmental and Social Epidemiology Unit, National Institute of Health, 00161 Rome, Italy; marco.giustini@iss.it; 3 Multiple Sclerosis Unit, IRCCS Fondazione Santa Lucia, 00179 Rome, Italy; s.catani@hsantalucia.it; 4 Department of Biomedical and Dental Sciences and Morphofunctional Imaging, University of Messina, 98122 Messina, Italy; stm_mondello@hotmail.com; 5 Experimental Neuro-Psychobiology Laboratory, IRCCS Fondazione Santa Lucia, 00179 Rome, Italy; i.piccolino@hsantalucia.it (I.P.); f.iannuzzi@hsantalucia.it (F.I.); 6 Department of Neurobiology, Center for Neurotrauma, Multiomics & Biomarkers (CNMB), Neuroscience Institute, Morehouse School of Medicine, Atlanta, GE 30310, USA; kawangwang17@gmail.com; 7 Brain Rehabilitation Research Center (BRRC), Malcom Randall Veterans Affairs Medical Center, Gainesville, FL 32608, USA; 8 Center for Visual and Neurocognitive Rehabilitation, Atlanta VA Health Care System, Decatur, GA 30033, USA; 9 Banyan Biomarkers Inc., Alachua, FL 32615, USA

**Keywords:** acquired brain injury, prolonged disorders of consciousness, serum biomarkers

## Abstract

Introduction: Serum biomarkers, such as Neurofilament Light (NF-L), Glial Fibrillary Acidic Protein (GFAP), Ubiquitin C-terminal Hydrolase (UCH-L1), and Total-tau (T-Tau) have been proposed for outcome prediction in the acute phase of severe traumatic brain injury, but they have been less investigated in patients with prolonged DoC (p-DoC). Methods: We enrolled 25 p-DoC patients according to the Coma Recovery Scale-Revised (CRS-R). We identified different time points: injury onset (t_0_), first blood sampling at admission in Neurorehabilitation (t_1_), and second blood sampling at discharge (t_2_). Patients were split into improved (improved level of consciousness from t_1_ to t_2_) and not-improved (unchanged or worsened level of consciousness from t_1_ to t_2_). Results: All biomarker levels decreased over time, even though each biomarker reveals typical features. Serum GFAP showed a weak correlation between t_1_ and t_2_ (*p* = 0.001), while no correlation was observed for serum NF-L (*p* = 0.955), UCH-L1 (*p* = 0.693), and T-Tau (*p* = 0.535) between t_1_ and t_2_. Improved patients showed a significant decrease in the level of NF-L (*p* = 0.0001), UCH-L1 (*p* = 0.001), and T-Tau (*p* = 0.002), but not for serum GFAP (*p* = 0.283). No significant statistical differences were observed in the not-improved group. Conclusions: A significant correlation was found between the level of consciousness improvement and decreased NF-L, UCH-L1, and T-Tau levels. Future studies on the association of serum biomarkers with neurophysiological and neuroimaging prognostic indicators are recommended.

## 1. Introduction

Disorders of Consciousness (DoC) are characterized by alterations in arousal and/or awareness, often caused by severe acquired brain injury (sABI) [1], and comprise different clinical states, including coma [2], Unresponsive Wakefulness Syndrome (UWS) [3] also known as Vegetative State (VS) [4], and Minimally Consciousness State (MCS) [5]. They are defined as “prolonged” when a DoC lasts more than 28 days [6,7]. UWS is characterized by a complete lack of self- and external world awareness, where patients recover spontaneous eye-opening and sleep-wake cycles [3]. MCS, which may follow coma or UWS, is identified by the reappearance of different degrees of awareness [8], and it has been classified into (i) MCS minus (MCS−), in which patients show non-reflex responses, such as localizing noxious stimuli or visual pursuing of a moving object/person or emotional reactions to salient stimuli and (ii) MCS plus (MCS+), where they show intelligible verbalization, yes/no responses, or command-following [9,10]. Patients showing functional object use or accurate functional communication are considered as having emerged from MCS (exit MCS/e-MCS) [11]. The continuous evolution of knowledge within the spectrum of DoC arises from the growing necessity of clarifying the neuropathological overlap among UWS and other clinical conditions, such as MCS and locked-in syndrome (LIS) [12].

The lack of evidence on the physiopathological mechanisms underlying sABI with DoC makes it difficult to promote practice standards or guidelines for a specific pharmacotherapy, and only treatment options (lowest level of recommendation) are currently available [13,14,15,16,17]. Therefore, physicians rely on clinical experience and treatment options, often facing the choice of prescribing off-label drugs [18]. Immune dysregulation has often been reported in sABI. The immediate and intense endogenous neuroinflammatory response after brain injury, which is originally aimed at defending and repairing the central nervous system (CNS), has been suggested to be involved in the development of secondary brain damage and adverse outcomes [19]. This inflammatory response is largely driven by cytokines and increased levels of inflammatory agents within the injured brain, including tumor necrosis factor (TNF)-α, interleukin (IL)-1β, IL-6, and intercellular adhesion molecule 1(ICAM-1), are believed to contribute to the overall cerebral damage [20,21,22]. In particular, Interleukin-18 (IL-18) has been identified to be persistently elevated after traumatic brain injury (TBI) [23], and severe TBI patients in the post-acute rehabilitation time period have increased serum levels of IL-18 in comparison with healthy control subjects [24]. Also, microRNAs (miRNAs), involved in gene regulation, may affect recovery in patients with p-DoC. A recent study [25] investigated the role of five miRNAs (i.e., 150-5p, 132-3p, 23b-3p, 451a, and 16-5p) in a cohort of 30 p-DoC patients, suggesting that miRNAs 132-3p and 23b-3p may serve as prognostic biomarkers, improving therapeutic interventions in this clinical population. Immunodepression was also observed in patients after prolonged coma following head injury, and together with the increase of serum cortisol levels and medical complications, it may contribute to the final outcome of medical complications [26,27,28]. Additionally, some studies provided the first evidence that serum levels of Microtubule-associated protein (MAP-2), a protein expressed selectively in neurons [29], can be tracked 6 months after injury in subjects who suffered from severe TBI supporting its role as a potential marker for emergence to higher levels of functional recovery [30].

Overall, numerous blood and cerebrospinal fluid (CSF) biomarkers have been proposed for severity assessment and outcome prediction in the acute phase of severe TBI. However, they have been less investigated in patients with prolonged DoC (p-DoC), regardless of their potential value as clinical predictors in this context [31]. Blood is a more convenient and less invasive source of biomolecules than CSF. Furthermore, correlations between blood markers and those found in CSF in patients with brain damage have been extensively studied in various neurological conditions, including TBI, stroke, and neurodegenerative diseases [32,33]. The development of ultrasensitive technologies has allowed the reliable quantification of very low blood biomarker concentrations, which may help clinicians define and better understand underlying persistent pathobiological mechanisms after TBI that may lead to a range of adverse psychiatric sequelae (e.g., psychosis and, in particular, schizophrenia) [34] or trigger persistent neurodegeneration [35], that, in turn, further affect the chance of recovering consciousness and worsen functional outcomes. One such marker is microtubule-associated protein Tau [or total Tau and list hyperphosphorylated form (P-Tau)]. Serum Total Tau (T-tau) is one of the most well-characterized biomarkers for severe TBI [36,37,38], showing high increases within hours of the injury [37]. In this context, serum biomarkers provide a good opportunity to better characterize ongoing brain degeneration, as such information cannot be obtained at the same level of detail with neurophysiological or neuroimaging techniques [31]. Consequently, the identification of new blood-based biomarkers is crucial in the diagnosis of the p-DoC and for the neurological outcome prediction after severe brain injury [39]. Coppola et al. [40] evaluated the brain-derived neurotrophic factor protein (BDNF) and the soluble cell adhesion molecules proteins (CAMs) in a sample of p-DoC patients by identifying BDNF as a possible blood marker for the diagnosis of pDoC and highlighting that soluble Neural CAM (Ncam) protein could find useful applications in the clinical evolution of the pDoC since it can discriminate between the VS/UWS subgroup compared to the MCS subgroup.

The ABI-related protein blood biomarkers mostly investigated in recent years [41,42,43] are Neurofilament Light (NF-L), Ubiquitin C-terminal Hydrolase (UCH-L1), T-tau, and Glial Fibrillary Acidic Protein (GFAP). In particular, NF-l and T-tau have emerged as potential markers of neurodegeneration [43,44]. Persistent elevated circulating NFL and tau levels have been detected up to 1 year after TBI [37,45], and NFL levels assessed at 8 months have been shown to predict diffusion tensor imaging metrics at >5 years after injury [46,47]. Glial fibrillary acidic protein (GFAP), a glial cytoskeletal protein, and ubiquitin C-terminal hydrolase-L1 (UCH-L1), a cytosolic neuronal protein, indicative of astroglial pathology and neuronal injury or death, respectively, have been extensively assessed in acute and subacute TBI and found to be correlated with injury severity and clinical outcomes [48,49]. In addition, GFAP and UCH-L1 as a tandem blood test have been cleared by both the Food and Drug Administration (FDA) and European Medicines Agency as an acute blood test to aid in the detection of intracranial lesions in mild to moderate TBI patients [50]. Nonetheless, their role in patients with p-DoC has not been fully examined. They may serve as complementary markers, providing independent information on the degree of underlying damage and the related but different pathobiological processes underpinning this patient population’s complex and heterogeneous condition.

Understanding the circuit mechanisms associated with the recovery of consciousness in patients with p-DoC, also in the post-acute and chronic phase, may open new directions for future research, allowing (i) the development of innovative diagnostic tools based on serum biomarkers [51], neuroimaging and electrophysiological measurements to guide longitudinal assessments of brain function [1,7,52,53,54] and (ii) the development of novel therapeutic interventions at the circuit and cellular level to aid consciousness recovery [55].

The present study aimed at investigating in a selected population of patients with p-DoC the possible p-DoC monitoring and the prognostic role of blood markers, such as NF-L, GFAP, UCH-L1, and T-Tau, poorly explored in this specific context.

## 2. Materials and Methods

### 2.1. Participants

We selected to focus on four brain injury-specific biomarkers, including astrogliosis/astroglial injury-linked GFAP neuronal cell body-linked UCH-L1, axonal injury markers such as NF-L and neurodegeneration-linked T-Tau, as these the most well established biofluid-based biomarkers following TBI [42].

Twenty-five sABI patients (mean age ± SD = 42.8 ± 17.3 years; M 68.0%, F 32.0%) admitted in a Neurorehabilitation Hospital in Rome (Italy) were consecutively enrolled for five years, according to the following inclusion criteria: (1) age ≥ 18 years; (2) diagnosis of sABI [56] (3) diagnosis of p-DoC [6,7], according to Coma Recovery Scale-Revised (CRS-R) [57]. The CRS-R [57] is a standardized scale for the neurobehavioral assessment of DoC patients consisting of 23 items split into 6 subscales. It is the gold standard scale for diagnosis, monitoring consciousness recovery, predicting outcomes, and assessing treatment effectiveness in this clinical population.

Exclusion criteria were: (1) patients in e-MCS; (2) patients with neurodegenerative and progressive neuroinflammatory disorders, such as multiple sclerosis; (3) patients with previous psychiatric disorders and substance abuse (alcohol, minor and major drugs).

Three different time points, labeled as “t”, were identified: injury onset (t_0_), first blood sampling at admission in Neurorehabilitation (t_1_), and second blood sampling at discharge (t_2_). The timing of the blood samples changed because collecting them during medical complications or infections that required anti-inflammatory and antibiotic therapies was rigorously avoided. All the blood samples were collected in the morning, before the administration of drugs acting on the CNS (e.g., sedatives, antidepressants, anti-epileptics, GABAergics, pain killers, anti-spasticity, dopaminergic, etc.).

Demographic and clinical data (e.g., unconsciousness duration, comorbidities, etc.) and the time intervals between each time point were collected for all sABI patients.

Disability Rating Scale (DRS) [58], a functional disability tool mainly used to detect and measure clinical changes in individuals who have sustained severe or moderate traumatic brain injury, was obtained at t_1_ and t_2_. Also, CRS-R was recorded at t_1_ and t_2_ to assess the possible consciousness recovery between the two time points.

Socio-demographic and clinical data of p-DoC patients at t_1_ (i.e., first blood sampling) are shown in Table 1 and Table 2, respectively.

The study was approved by the local Ethics Committee of IRCCS Fondazione Santa Lucia, Rome, Italy (Protocol number: CE-AG4-Prog.72-23) and performed according to the ethical principles introduced in 1964 by the Declaration of Helsinki and its later amendments. Legal representatives of the patients signed informed consent.

### 2.2. Biomarkers Analysis

The emerging neurology biomarkers relevant for traumatic brain injury, GFAP, UCH-L1, T-Tau, and NF-L, were measured in serum with the Quanterix Single Molecular Array (SIMOA) Human Neurology 4-Plex assay (Quanterix, Lexington, MA, USA). The Quanterix Simoa N4PB kit measures GFAP, UCH-L1, T-Tau, and NF-L serum concentrations on a multiplex array simultaneously, according to manufacturer’s instructions https://www.quanterix.com/products-technology/assays/neuro-4-plex-b, accessed on 15 September 2023.

Blood samples were collected via venepuncture in a serum collecting tube, left to clot for 30–60 min, and centrifuged at 1000× *g* for 15 min before storage at −80 °C and temperature-controlled shipment on dry ice for subsequent analysis. For a detailed description of the assay methodology, please consult Korley et al., 2018 [59]. Assays were run at the University of Florida’s Program for Neurotrauma, Neuroproteomics and Biomarkers Research facility.

Additional details on assay performance are available at: https://www.quanterix.com/wp-content/uploads/2020/12/N4PB-Data-Sheet_Rev02.pdf, accessed on 15 September 2023.

Serum samples were run at 1/8 dilution with Quanterix sample diluent. Thus, the reported values were adjusted by multiplying by a factor of eight. The lower Limit of Quantification (LLOQ) for NF-L, GFAP, UCH-L1, and Tau were 0.500, 9.38, 9.39, and 0.124 pg/mL, respectively. The limit of Detection (LOD) for NF-L, GFAP, UCH-L1, and Tau were 0.105, 1.51, 1.90, and 0.0408 pg/mL, respectively, and the assay range for NF-L, GFAP, UCH-L1, and Tau were 0–2000, 0–40,000, 0–40,000, and 0–400 pg/mL, respectively. Samples with one or more analyte (i.e., biomarker) levels above the aforementioned ranges were further diluted (e.g., 1/16) to make the values fall within the range. Analyte values that were below LOD were reported as ½ of the analyte’s LOD (e.g., a low value of NF-L was reported as ½ × 0.105 = 0.0525 pg/mL). Intra-assay % Coefficient of Variation (%CV) for NF-L, GFAP, UCH-L1 and T-Tau were 3.3–7.6%, 4.1–13.4%, 3.3–14.7%, 2.4–7.3%, respectively, while inter-assay %CV for NF-L, GFAP, UCH-L1 and Tau were 5.4–10.2%, 4.7 = 9.2%, 5.6–7.9%, 3.8–7.1%, respectively [60].

### 2.3. Statistical Analysis

The serum biomarker values were significantly skewed and were, therefore, log-transformed for analyses, except where otherwise specified. As the biomarkers showed values on different scales, they were also standardized to make comparisons between them possible.

According to CRS-R scores, patients were split into two groups: (i) improved (i.e., if they showed an improved level of consciousness from t_1_ to t_2_, and (ii) not improved (unchanged or worsened level of consciousness from t_1_ to t_2_.

Comparisons were performed using the Mann-Whitney U test for unmatched data or the Wilcoxon matched-pairs signed-rank test. Spearman’s ρ (Rho) with Bonferroni correction has also been run. We assumed that the correlation was: (a) very high if ρ ≥ 0.90; (b) high if 70 ≤ ρ < 90; (c) moderate if 50 ≤ ρ < 70; (d) low if 30 ≤ ρ < 50; (e) negligible if 0 ≤ ρ < 30″ [61]. Kendall’s τ (tau) was used to estimate the correlation between paired observations. The same cut-offs were used for Kendall’s τ as for Spearman’s ρ. Consequently, the interpretation of the τ statistic was the same as the ρ statistic [62,63]. The coefficient of determination (r^2^) has been used in a regression model to determine the proportion of variance in the dependent variable explained by the independent variable. Continuous data were presented as mean and standard deviation (sd) or median and interquartile range (IQR), as appropriate. All statistical tests were one-sided with 95% confidence intervals. The Clopper–Pearson “exact” method for calculating binomial confidence intervals was used [64]. Any *p*-value < 0.05 was considered as statistically significant.

Statistical analysis was performed using STATA Stata/SE 15.1 (StataCorp, College Station, TX, USA).

## 3. Results

Regarding the etiology of sABI, 10 out of 25 patients (40.0%) had TBI, whilst 15 (60.0%) were classified as non-TBI due to hemorrhage, ischemia, and subarachnoid hemorrhage.

Even though the sample was mainly composed of males, we did not find a statistically significant difference between the two groups (i.e., improved and not improved patients) regarding to gender (*p* = 0.190). Conversely, a statistically significant difference was observed between TBI and non-TBI patients: the first group (i.e., TBI) showed a significantly lower percentage of females (10.0% vs. 46.7%, *p* = 0.054) in comparison to the second one.

TBI patients showed a significantly lower median age compared to non-TBI patients (25.3 years vs. 48.0 years, *p* = 0.0042) as well as longer coma length (median: 31.5 days vs. 15.0 days; *p* = 0.0516), and, as aforementioned, a lower percentage of females (10.0% vs. 46.7%, *p* = 0.054). TBI and non-TBI patients had no statistically significant difference in DRS and CRS-R scores at t_1_ and t_2_, respectively, as well as in the time interval between t_0_ and t_1_ and between t_1_ and t_2_ (see Table 3).

Overall, the time interval between t_0_ and t_1_ ranged from 40 to 734 days (mean: 197.0 days; sd: ±150.4 days), while the time interval between t_1_ and t_2_ ranged from 7 to 509 days (mean: 219.3 days; sd: ±156.7 days).

According to CRS-R, at t_1_ six out 25 patients (24.0%) were diagnosed as UWS, 10 (40.0%) as MCS−, 8 (32.0%) were classified as MCS+, and only 1 patient (4.0%) was diagnosed as LIS, a syndrome considered as a recovery phase from DoC [65] Fourteen patients (56.0%) improved their responsiveness, 9 participants showed an unchanged level of consciousness, and 2 worsened their clinical condition between t_1_ and t_2_, being diagnosed as UWS. No statistically significant difference between the two groups (improved and not improved patients) was observed regarding: (i) age (*p* = 0.142), (ii) gender (*p* = 0.190), (iii) coma length (*p* = 0.054), (iv) etiology (*p* = 0.010) and (v) CRS-R at t_2_ (*p* = 0.112).

Overall, CRS-R scores increased between t_1_ and t_2_: the CRS-R median values were 10 (IQR 4) and 21 (IQR 15) at t_1_ and t_2_, respectively. According to the Wilcoxon signed-rank test, the distributions of CRS-R scores are statistically different (*p* = 0.0013) between t_1_ and t_2_.

As displayed in Table 4, GFAP showed higher levels in UWS patients at t_1_ (*p* = 0.037), as well as all four biomarkers at t_2_.

Serum GFAP showed a weak correlation between t_1_ and t_2_ (Kendall’s τ = 0.498; *p* = 0.001), while no correlation was observed for serum NF-L (Kendall’s τ = −0.0130; *p* = 0.955), UCH L1 (Kendall’s τ = 0.0649; *p* = 0.693), and T-Tau (Kendall’s τ = 0.099; *p* = 0.535) between t_1_ and t_2_.

**Figure 1:**
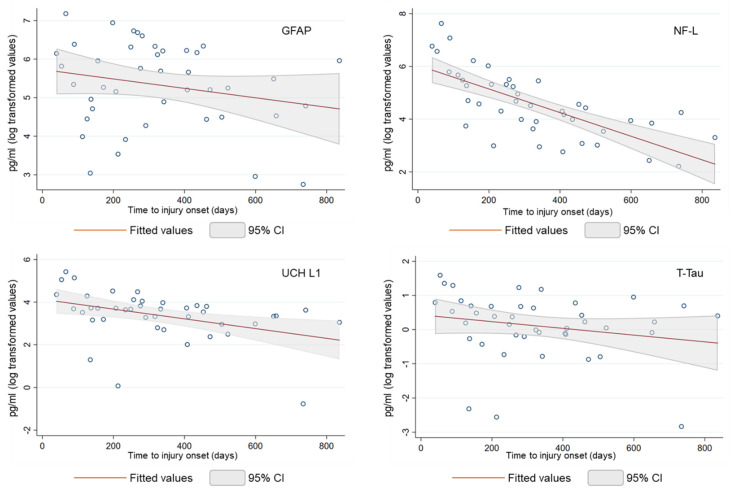
Biomarker levels (in log pg/mL) showed by patients at t_1_ and t_2_.

Due to the strong skew of the serum biomarker concentrations, Figure 1 shows the log-transformed biomarker levels for both all patients and blood sampling. NF-L, UCH L1, and T-Tau were statistically independent between t_1_ and t_2_, whilst GFAP showed a weak correlation between the two blood samplings. To better represent the evolution of biomarker values over time, the serum biomarker values were considered together at t_1_ and t_2_. All biomarker levels decreased over time, even though each biomarker reveals typical features and distinct temporal dynamics.

NF-L decreased over the study period (r^2^ = 0.513), with a significant decrease in the first 100 days from the injury onset (−89% compared to average of the serum level at a time length ≥ 100 days from t_0_), while the serum GFAP time course was variable (r^2^ = 0.050), with no time effect on biomarker concentrations (−49% compared to average of the serum level at a time length ≥ 100 days from t_0_). Serum UCH L1 concentrations showed a monotonic pattern similar to the NF-L biomarker (−75% compared to an average of the serum level at a time length ≥ 100 days from t_0_). However, three outlier measurements reduced the value of the coefficient of determination (r^2^ = 0.163). Lastly, serum T-Tau showed a variable trend over time (r^2^ = 0.044), with a less marked decrease during the first 100 days (−60% compared to an average of the serum level at a time length ≥ 100 days from t_0_).

At t_1_, the levels of NF-L, UCH L1, and T-Tau were significantly correlated with each other (Table 5a), but the strength of these correlations decreased at t_2_ (Table 5b), with the exception of the T-Tau biomarker, preserving its good correlation with UCH L1. No significant correlation between biomarkers and age, gender, etiology, DRS, and CRS-R has been observed (Table 5b), although CRS-R showed a moderate correlation with NF-L (ρ = −0.515), UCH L1 (ρ = −0.564) and T-Tau (ρ = −0.608) at t_2_.

TBI patients showed a significant decrease in the level of NF-L (*p* = 0.0078) and UCH L1 (*p* = 0.0039) between t_1_ and t_2_, but not for serum GFAP (*p* = 0.3594) and T-Tau (*p* = 0.7344). Non-TBI patients showed a significant decrease only in the level of NF-L (*p* = 0.0081).

**Figure 2:**
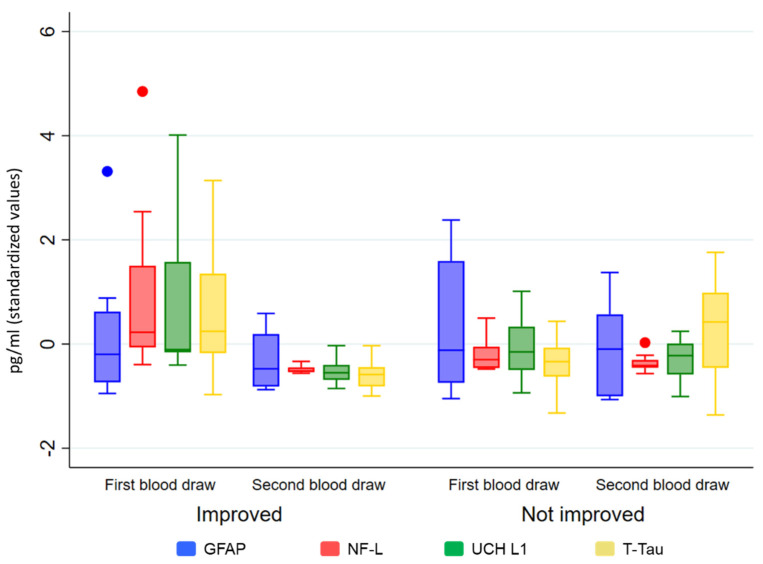
Biomarker levels (in standardized values) by t_1_ and t_2_ and groups (improved/not improved).

Patients who improved their level of consciousness (i.e., improvement in CRS-R scores) between t_1_ and t_2_ showed a significant decrease in the level of NF-L (*p* = 0.0001), UCH L1 (*p* = 0.001), and T-Tau (*p* = 0.002), but not for serum GFAP (*p* = 0.283). No significant statistical differences were observed in the group that had not improved (see Figure 2).

## 4. Discussion

Behavioral assessments of p-DoC patients could not be sufficient for a correct diagnosis and prognosis [39], but a multidisciplinary approach, combining data obtained from clinical evaluation and neuroimaging, could aid clinicians to improve diagnosis and follow the recovery trajectory in this clinical population [66,67]. However, advanced neuroimaging cannot be often applied or not applied in a large number of patients due to technical issues in acquisition and analysis [68,69].

In patients with p-DoC, neurophysiological indicators have also shown some interesting predictive efficacy [52,70,71,72,73]. Accordingly, the study and the introduction of new serum biomarkers are pivotal in the diagnosis and/or prognosis of patients with p-DoC. Indeed, newer biomarkers, such as T-Tau and NF-L, have been revealed to be crucial for the evaluation of neuronal injury of acute DoC patients and, more specifically, have shown a higher specificity for brain injury due to cardiac arrest and are being studied [66].

We examined the role and the evolution of blood concentrations of serum markers, such as NF-L, GFAP, UCH-L1, and T-Tau, in a selected population of patients with p-DoC in the post-acute and chronic phase (ranging from 1 to 24 months from brain damage).

The results showed that all biomarker levels decreased over time, even though each biomarker reveals typical features and distinct temporal dynamics. A significant correlation was found between the level of consciousness improvement and decreased NF-L, UCH-L1, and T-Tau levels, whilst serum concentrations of GFAP marker seem to not be associated with the recovery of consciousness, suggesting a different trend of this biomarker candidate in monitoring the disease state, possibly linked to a complex role of glial injury in the progression of the consciousness disorder. The serum NF-L concentration suddenly lapsed, with a significant decrease in the first 100 days from the injury onset. Between 400 and 500 days from the injury onset, its mean blood concentration was 94% lower than that found in the first 100 days from injury. Serum UCH L1 concentrations showed a pattern similar to the NF-L biomarker (−75% compared to an average of the serum level at a time length ≥ 100 days from t_0_), while the serum GFAP trend was variable and high over time, with no time effect on biomarker concentrations. Indeed, between 400 and 500 days from the injury onset, the GFAP mean blood concentration was 44% lower compared to the one found in the first 100 days from injury. Lastly, serum T-Tau, which, as mentioned above, appears related to the level of consciousness improvement, showed a less marked decrease during the first 100 days (−60% compared to an average of the serum level at a time length ≥ 100 days from t_0_), since the T-Tau mean blood concentration, between 400 and 500 days from the injury onset, was roughly half that one of the first 100 days. While the NF-L and UCH L1 concentrations seem to be related to the time from injury, the T-Tau concentration appears to be less time-dependent, suggesting a T-Tau potential prognostic role in detecting the recovery of consciousness in p-DoC patients.

The difference in biomarker temporal dynamics between the patient groups may be attributable to the different cell origin and protein characteristics, as well as to the distinctive pathophysiology and tissue damage associated with different etiology which may affect the passage across the blood-brain barrier (BBB), thereby resulting in distinct biomarker-specific release patterns [74,75,76]. A recent study demonstrated that biomarker levels and time course are associated with the overall magnitude of injury, BBB disruption severity, and different types of injuries and locations [77].

The major strength of this study was the assessment and monitoring of possible levels of consciousness changes in the post-acute phase in a selected population of patients with p-DoC by a longitudinal approach.

However, our study had some limitations. Firstly, the small sample size and mixed etiology did not allow any generalization, and additional studies with larger patient populations are needed to establish the prognostic value of serum biomarkers in patients with p-DoC. Secondly, the lack of a healthy control group does not allow for target values of serum concentrations, and comparing the evolution of observed concentrations limits the generalizability of our findings by suggesting that we interpret our results with caution. Additionally, non-standardized temporal distances neither between the injury onset and blood samplings nor between the two blood samplings can affect the interpretation of our findings, especially those related to changes in serum biomarkers, which seem to be more time-dependent. Finally, the lack of long-term follow-up of patients with p-DoC may represent a limit to our preliminary results regarding the final outcome of the patient population. Future studies on the associations of serum biomarkers with neurophysiological and neuroimaging prognostic indicators may be of some interest.

Despite these limitations, this preliminary study suggests a possible prognostic role of some blood markers, such as NF-L, GFAP, UCH-L1, and T-Tau in patients with p-DoC, mainly highlighting the potential role of T-Tau in detecting the recovery of consciousness in this clinical population.

## 5. Conclusions

Our study aimed at investigating in a selected population of patients with p-DoC the prognostic role of blood markers poorly explored in this specific context by revealing a correlation between the level of consciousness improvement and decreased NF-L, UCH-L1, and T-Tau levels.

The major strength of this study was the assessment and monitoring of possible levels of consciousness changes in the post-acute phase in a selected population of patients with p-DoC by a longitudinal approach. However, the study was conducted under some constraints, such as the non-standardized temporal distances neither between the injury onset and blood samplings nor between the two blood samplings and the lack of long-term follow-up of patients with p-DoC.

Further larger studies are needed in order to make our preliminary results more reliable and to investigate the T-Tau utility as a biomarker deeply to assess the possible changes in consciousness levels and to monitor outcomes in p-DoC patients for improving their treatment and management. Future studies on the association of serum biomarkers with neurophysiological and neuroimaging prognostic indicators are also recommended.

## Figures and Tables

**Table 1:** Patient socio-demographic data at t_1_.

Socio-Demographic Data	Values
Gender (M; %)	68%
Age (yrs; ±sd)	42.8 ± 17.28
Educational level (%)	
University degree	20%
High School diploma	32%
Lower secondary school diploma	48%

**Table 2:** Patient clinical data at t_1_.

Clinical Data	Values
Coma length in days (mean ± sd)	28.1 ± 15.00
Time since injury in days (mean ± sd)	197 ± 150.45
Aetiology (%)	
TBI	40%
non-TBI	60%
Diagnosis (%)	
UWS	24%
MCS−	40%
MCS+	32%
LIS	4%
Percutaneous Endoscopic Gastrostomy or Nasogastric Tube (%)	88%

**Table 3:** Comparison between TBI and non-TBI patients by DRS and CRS-R.

	TBI Patients	Non TBI Patients	*p* (2 Sided)
Age (Years, median)	25.3	48.0	0.0042
Gender (% Female)	10.0%	46.7%	0.0540
DRS at t_1_ (median)	22	21	0.7481
DRS at t_2_ (median)	18.5	21	0.1710
CRS-R at t_1_ (median)	10.5	10	1.0000
CRS-R at t_2_ (median)	21	21	0.5583
Coma length (days, median)	31.5	15	0.0516
Time interval between t_0_ and t_1_ (days, median)	136	156	0.6830
Time interval between t_1_ and t_2_ (days, median)	303.5	176.5	0.0858

**Table 4:** Biomarker levels (in pg/mL) in MCS e UWS patients at t_1_ e t_2_.

	t_1_			t_2_		
	MCS (Median)	UWS (Median)	*p* (One Side)	MCS (Median)	UWS (Median)	*p* (One Side)
GFAP	209.244	677.673	0.037	186.062	488.717	0.009
NF-L	240.041	199.255	0.354	36.107	101.809	0.004
UCH L1	39.785	51.191	0.304	21.503	45.963	0.000
T-Tau	1.704	1.008	0.088	0.906	2.074	0.001

**Table 5:** (**a**) Correlation between biomarkers and CRS-R at t_1_. (**b**) Correlation between biomarkers and CRS-R at t_2_.

**(a)**									
	**Age**	**Gender**	**Aetiology**	**DRS**	**CRS-R**	**GFAP**	**NFL**	**UCH L1**	**T-Tau**
Age	1.000								
Gender	0.186	1.000							
Aetiology	0.448	0.428	1.000						
DRS	−0.356	−0.168	0.024	1.000					
CRS-R	0.177	0.119	−0.249	−0.737	1.000				
GFAP	−0.255	0.330	0.319	0.181	−0.404	1.000			
NF-L	−0.152	0.248	0.123	−0.232	0.036	0.572	1.000		
UCH L1	−0.283	0.014	−0.016	−0.103	−0.179	0.681	0.837	1.000	
T-Tau	−0.119	0.303	−0.052	−0.485	0.223	0.510	0.842	0.660	1.000
**(b)**									
	**Age**	**Gender**	**Aetiology**	**DRS**	**CRS-R**	**GFAP**	**NFL**	**UCH L1**	**T-Tau**
Age	1.000								
Gender	0.209	1.000							
Aetiology	0.483	0.406	1.000						
DRS	−0.010	0.067	0.313	1.000					
CRS-R	−0.097	0.109	−0.232	−0.872	1.000				
GFAP	0.039	0.551	0.496	0.405	−0.334	1.000			
NF-L	0.478	0.372	0.539	0.426	−0.515	0.562	1.000		
UCH L1	0.249	0.045	0.334	0.521	−0.564	0.513	0.689	1.000	
T-Tau	0.079	0.000	0.113	0.577	−0.608	0.362	0.528	0.664	1.000

## Data Availability

The data are not publicly available due to [containing information that could compromise the privacy of research participants].

## Abbreviations

BBB	Blood Brain Barrier
BDNF	Brain-derived neurotrophic factor
CAMs	Cell adhesion molecules protein
CNS	central nervous system
CRS-R	Coma Recovery Scale-Revised
CSF	cerebrospinal fluid
CV	Coefficient of Variation
Doc	Disorders of Consciousness
DRS	Disability Rating Scale
e-MCS	exit MCS
FDA	Food and Drug administration
GFAP	Glial Fibrillary Acidic Protein
ICAM-1	intercellular adhesion molecule 1
IL	interleukin
IQR	interquartile range
kDa	kilodalton
LIS	locked-in syndrome
LLOQ	Lower Limit of Quantification
LOD	Limit of Detection
MAP	Microtubule-associated protein
MCS	Minimally Consciousness State
MicroRNAs	miRNAs
NF-L	Neurofilament Light
p-DoC	prolonged Disorders of Consciousness
sABI	severe Acquired Brain Injury
TBI	traumatic brain injury
TNF	tumor necrosis factor
T-Tau	Total Tau
UCH-L1	Ubiquitin C-terminal Hydrolase
UWS	Unresponsive Wakefulness Syndrome
VS	Vegetative State
